# Supplementary material for: Rats Selected for Different Nervous Excitability: Long-Term Emotional–Painful Stress Affects the Dynamics of DNA Damage in Cells of Several Brain Areas
Source: Int J Mol Sci. 2024 Jan 13;25(2):994. doi: 10.3390/ijms25020994 (PMC10815859; doi:10.3390/ijms25020994)
Supplement: Supplementary file 1 [file ijms-25-00994-s001.zip › ijms-2800125-supplementary.pdf]

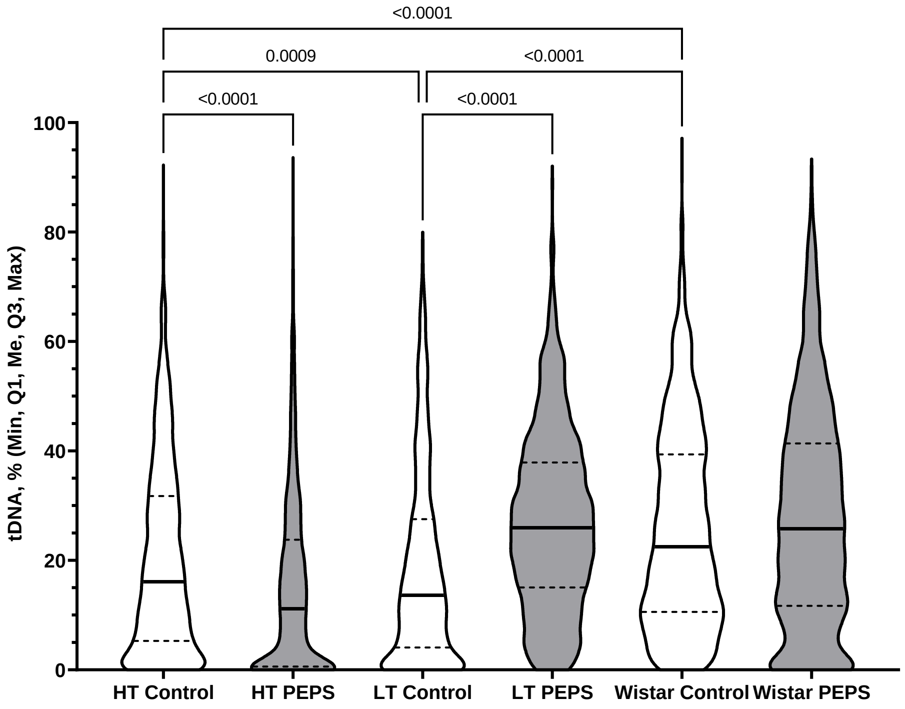

Supplementary Figure S1. tDNA content in PFC cells of unstressed (Control) and stressed (2 hours after PEPS) rats of three strains (HT, LT, and Wistar). Significant differences in pairwise comparison (determined in Kruskal-Wallis test) between stressed and the corresponding control groups as well as differences between unstressed animals of different strains are shown as lines with *p*-level.

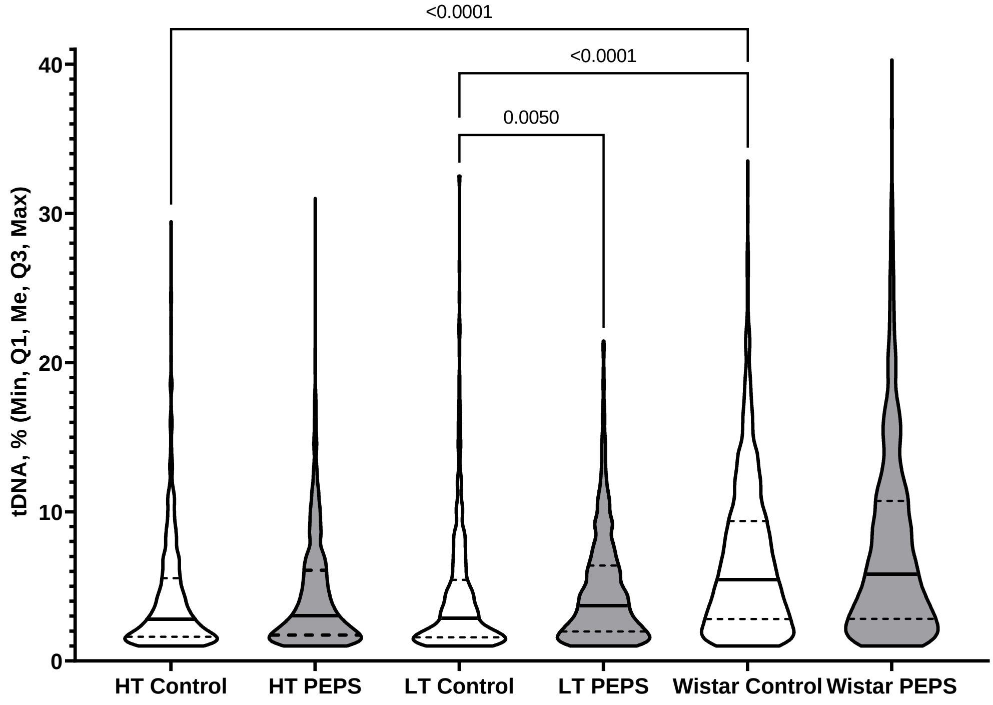

Supplementary Figure S2. tDNA content in PFC cells of unstressed (Control) and stressed (2 weeks after PEPS) rats of three strains (HT, LT, and Wistar). Significant differences in pairwise comparison (determined in Kruskal-Wallis test) between stressed and the corresponding control groups as well as differences between unstressed animals of different strains are shown as lines with *p*-level.

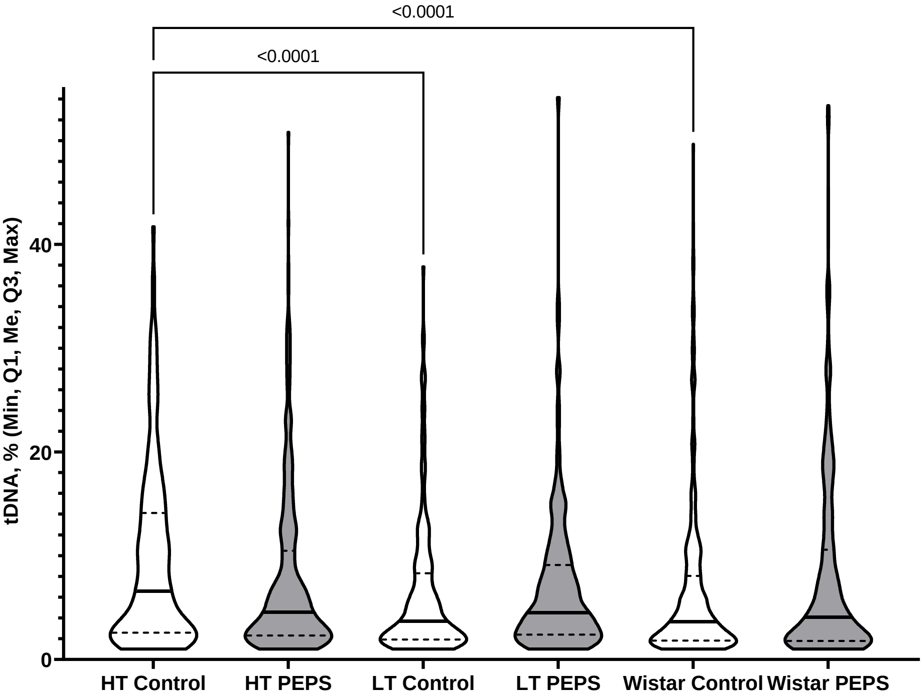

Supplementary Figure S3. tDNA content in PFC cells of unstressed (Control) and stressed (2 months after PEPS) rats of three strains (HT, LT, and Wistar). Significant differences in pairwise comparison (determined in Kruskal-Wallis test) between stressed and the corresponding control groups as well as differences between unstressed animals of different strains are shown as lines with *p*-level.

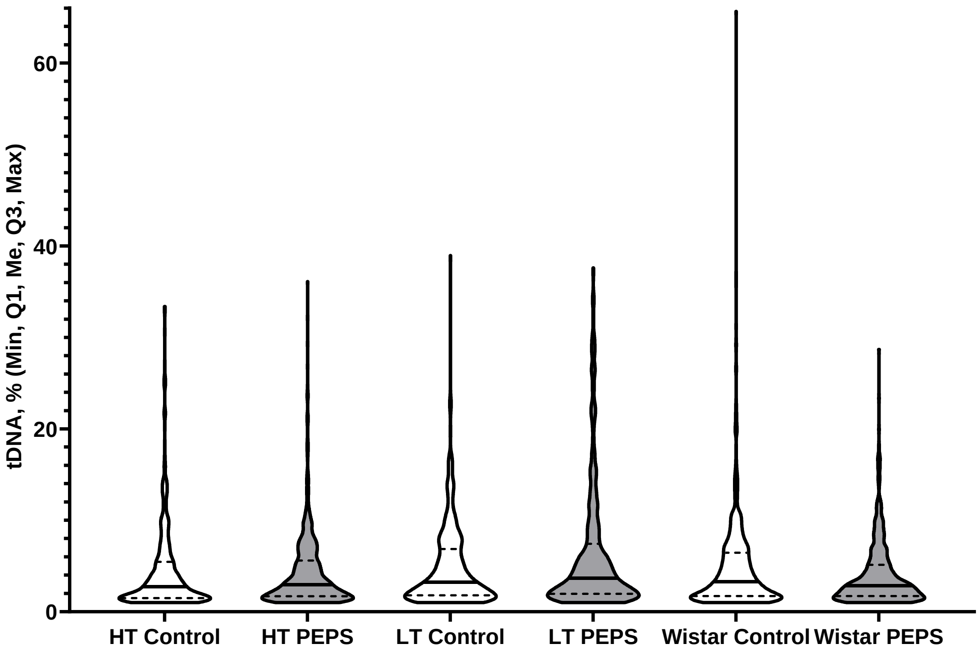

Supplementary Figure S4. tDNA content in hippocampal cells of unstressed (Control) and stressed (2 weeks after PEPS) rats of three strains (HT, LT, and Wistar). Significant differences in pairwise comparison (determined in Kruskal-Wallis test) between stressed and the corresponding control groups as well as differences between unstressed animals of different strains are shown as lines with *p*-level.

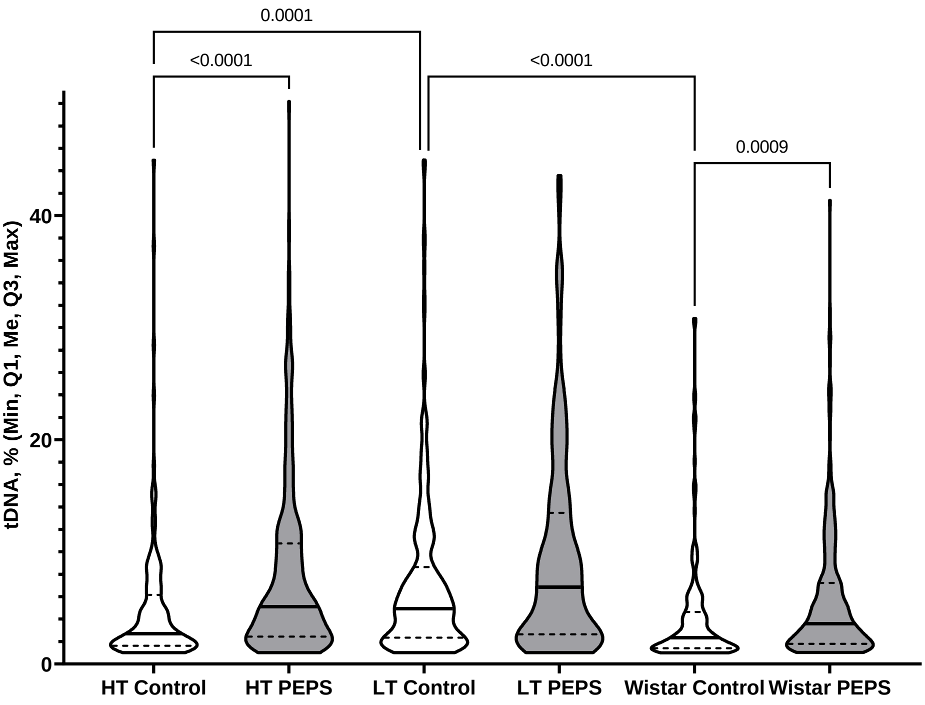

Supplementary Figure S5. tDNA content in hippocampal cells of unstressed (Control) and stressed (2 months after PEPS) rats of three strains (HT, LT, and Wistar). Significant differences in pairwise comparison (determined in Kruskal-Wallis test) between stressed and the corresponding control groups as well as differences between unstressed animals of different strains are shown as lines with *p*-level.

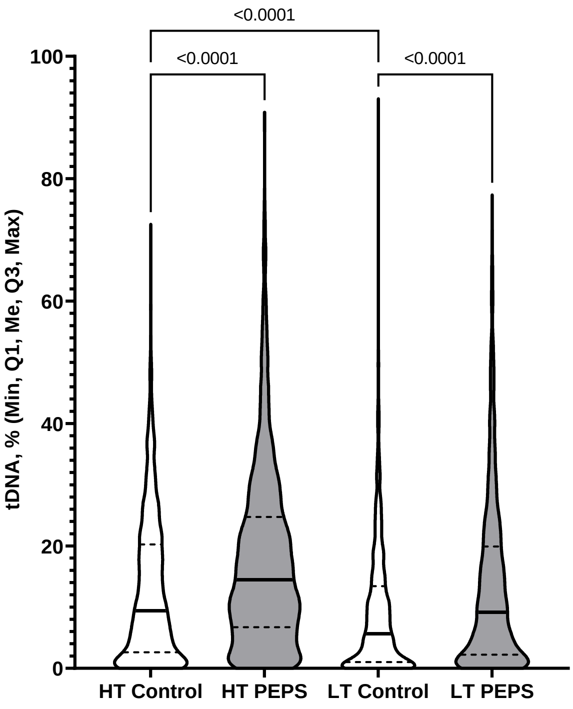

Supplementary Figure S6. tDNA content in amygdala cells of unstressed (Control) and stressed (1 hour after PEPS) rats of three strains (HT and LT). Significant differences in pairwise comparison (determined in Kruskal-Wallis test) between stressed and the corresponding control groups as well as differences between unstressed animals of different strains are shown as lines with *p*-level.

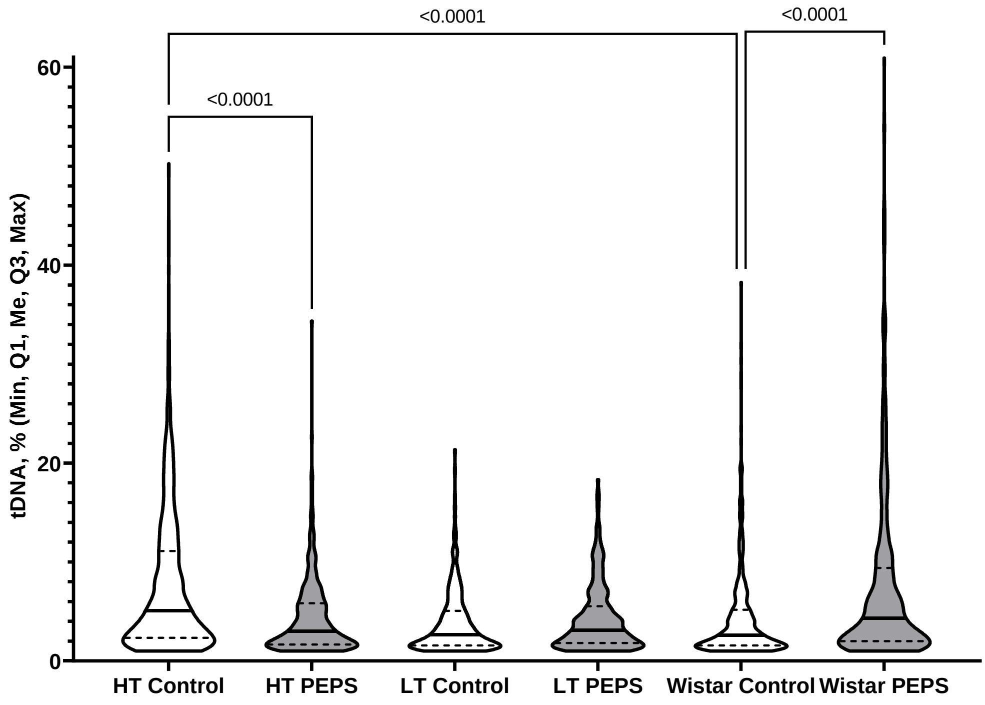

Supplementary Figure S7. tDNA content in amygdala cells of unstressed (Control) and stressed (2 weeks after PEPS) rats of three strains (HT, LT, and Wistar). Significant differences in pairwise comparison (determined in Kruskal-Wallis test) between stressed and the corresponding control groups as well as differences between unstressed animals of different strains are shown as lines with *p*-level.

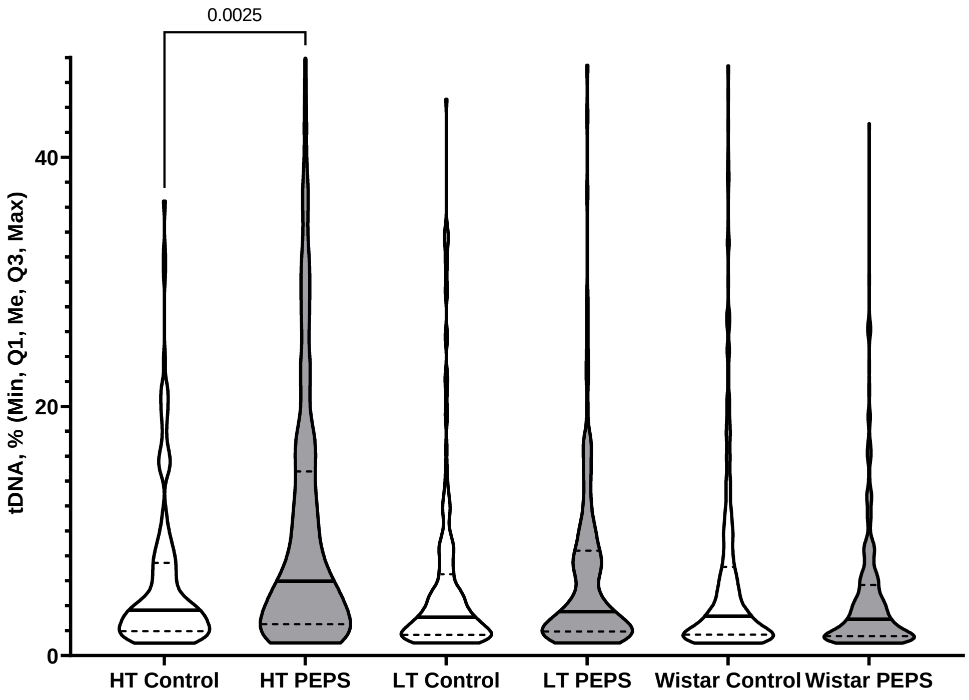

Supplementary Figure S8. tDNA content in amygdala cells of unstressed (Control) and stressed (2 months after PEPS) rats of three strains (HT, LT, and Wistar). Significant differences in pairwise comparison (determined in Kruskal-Wallis test) between stressed and the corresponding control groups as well as differences between unstressed animals of different strains are shown as lines with *p*-level.
